# The Voice of a Friend

**Published:** 1887-06

**Authors:** Belle Bush


### THE VOICE OF A FRIEND.

BY BELLE BUSH.

Oh, pleasant to me is the voice of a friend,
Whose thoughts and whose deeds unto harmony tend,
Whatever his station may be.

We're brothers and sisters, all children of God,
And, whether or not we have acres of sod,
We each can be happy and free.

We can speak a kind word, we can do a good deed,
And reap for our planting a harvest of seed ;
And that is the way to be free.

We can sing for the weary, can pray for the weak,
And jewels of truth for humanity seek ;
And thus shall we happiness see.

For happiness springs from each labor of worth,
And every good deed that we do upon earth,
The angels above us can see.

When cheerful and patient, when loving and mild,
We turn to each task with the trust of a child,
Then the white-winged watchers are nigh.

They know every thought, every beautiful deed,
And their love taketh note of whatever we need,
And lo ! ere we know it, 'tis nigh.

Sometimes it is pleasure, sometimes it is pain,
'Tis sunshine to-day, to-morrow 'tis rain,
'Tis best whatever may come :

For God on whose wisdom and bounty we call,
Embraces not one, but embraces us *all*
In a love that is leading us home.

—*Belvidere Seminary, New Jersey.*

